# Surgical resection and orbital iodine-125 brachytherapy for orbital malignancy: a novel treatment for orbital lymphoma

**DOI:** 10.1007/s10792-022-02594-x

**Published:** 2023-03-12

**Authors:** Nan Ma, Ping Wang, Shaobo Zhang, Xiaona Ning, Chenjun Guo, Qiong Zhang, Qilin Cheng, Jinbo Zhao, Yangjun Li

**Affiliations:** 1 Department of Ophthalmology, Tangdu Hospital, Air Force Medical University, No. 1 Xinsi Road, Xi’an, 710038 Shaanxi People’s Republic of China; 2 Department of Thoracic Surgery, Tangdu Hospital, Air Force Medical University, No. 1 Xinsi Road, Xi’an, 710038 Shaanxi People’s Republic of China

**Keywords:** Brachytherapy, Iodine-125, Orbital malignancy, Lymphoma, External radiation

## Abstract

**Objectives:**

Orbital lymphoma is one of the most common adult orbital malignancies, accounting for approximately 10% of all orbital tumors. This study aimed to analyze the effects of surgical resection and orbital iodine-125 brachytherapy implantation for orbital lymphoma.

**Patients and methods:**

This was a retrospective study. Clinical data of 10 patients were collected from October 2016 to November 2018 and followed up to March 2022. Patients underwent the primary surgery for maximal safe removal of the tumor. After a pathologic diagnosis of a primary orbital lymphoma was established, iodine-125 seed tubes were designed based on the tumor size and invasion range, and direct vision was placed into the nasolacrimal canal or/and under the orbital periosteum around the resection cavity during the secondary surgery. Then, follow-up data, including the general situation, ocular condition, and tumor recurrence, were recorded.

**Results:**

Of the 10 patients, the pathologic diagnoses included extranodal marginal zone lymphoma of mucosa-associated lymphoid tissue (6 cases), small lymphocytic lymphoma (1 case), mantle cell lymphoma (2 cases), and diffuse large B-cell lymphoma (1 case). The number of seeds implanted ranged from 16 to 40. The follow-up period ranged between 40 and 65 months. All patients in this study were alive and well had tumors that were completely controlled. No tumor recurrences or metastases occurred. Three patients had dry eye syndrome and two patients had abnormal facial sensation. No patient had radiodermatitis involving the skin around the eye, and no patient had radiation-related ophthalmopathy.

**Conclusions:**

Based on preliminary observations, iodine-125 brachytherapy implantation appeared to be a reasonable alternative to external irradiation for orbital lymphoma.

## Introduction

Lymphoma is the most frequent malignancy of the ocular adnexa [1]. Ocular adnexal lymphoma can arise in the conjunctiva, eyelids, orbit including the lacrimal gland [2]. Orbital lymphoma is one of the most common adult orbital malignancy, making up approximately 10% of all orbital tumors, 2% of all nodal and extranodal lymphomas [3–6], and 50–60% of ocular adnexal lymphomas [7]. Lymphomas are divided into 2 major categories, namely Hodgkin lymphoma (HL) and non-Hodgkin lymphoma (NHL). Both HL and the different NHLs can arise in the orbit [8].

In recent years, various treatment options have become available. The following criteria must be considered when treating orbital lymphoma: (A) subtype classification; (B) the extension of tumor and the presence or absence of metastases; (C) prognostic factors in relation to the patient and disease; and (D) the impact of the lymphoma on the eye and its function [9]. Generally, there are three primary treatments (surgery, chemotherapy, and external radiation), among which external radiation is considered the most common treatment for orbital lymphomas. Of note, external radiation has a variety of side effects, including serious cutaneous reactions, radiation cataracts, macular degeneration, retinopathy, unmitigated dry eye, and corneal ulceration secondary to xerophthalmia [10].

Implantation of interstitial radioactive sources, or brachytherapy, has been reported to exert good local control for solitary brain metastases [11, 12] and recurrent meningiomas [13–16]. Iodine-125 (^125^I) is the most commonly used source, with a half-life of 59 days [17]. ^125^I implantation and ^125^I plaque have been reported to yield good results in the treatment of ocular and orbital malignancies, including retinoblastoma, uveal and choroidal melanoma [18–20], adenoid cystic carcinoma of the lacrimal gland, orbital invasion by basal cell carcinoma, orbital extension of conjunctival, metastatic carcinoma [21], and orbital rhabdomyosarcoma [22]. Of note, there have been no prior reports of using ^125^I brachytherapy implantation as a treatment for orbital lymphoma. Herein, we provide a novel treatment involving ^125^I brachytherapy after surgical resection for orbital lymphoma.

## Materials and methods

### Patients

From October 2016 to November 2018, 10 patients diagnosed with orbital tumors underwent surgical resection in the Department of Ophthalmology at our hospital. The inclusion criteria were: (1) The bone marrow puncture results were normal; (2) systemic examination revealed no lymphoma at the other sites; (3) there was no malignant disease in the whole body; and (4) the vision of the affected eye was > 0.1. The exclusion criteria were: (1) The bone marrow puncture results were abnormal; (2) there was a lymphoma in sites other than the orbit; (3) the lesion involved multiple cranial nerves; (4) the patients had cachexia or could not tolerate the secondary surgery; and (5) women during pregnancy and lactation.

### Preparation, evaluation, and follow-up

After the pathologic diagnosis for the tumor specimen was established to be an orbital lymphoma, orbital ^125^I seed implantation was performed in the second surgery. Informed consent was obtained from all patients and approved by the Ethics Committee of the Hospital (No. 20170806). Tumor typing was assigned by pathologists using immunohistochemical methods. Patients were required to return to our institution for re-examination. Two weeks after implantation, wound healing was assessed. One month later, an orbital CT scan was performed to determine the exact location of the seeds. Three or six months later, patients underwent an orbital MRI or CT to assess the tumor for reduction or recurrence and a whole-body examination (ultrasound examination of cervical, supraclavicular, and abdominal lymph nodes) to check for tumor metastasis. Moreover, an ophthalmologic examination, including visual acuity, intraocular pressure, corneal and lens opacity, and fundus examination, was assigned at every postoperative re-examination. The patients were then evaluated at least twice yearly. For the diagnosis of dry eye, a tear secretion test and breakup time of tear film were applied. For the determination of dry eye, the results of the tear secretion test was < 10 mm, and the breakup time of the tear film was < 5 s. Abnormal facial sensation was recorded when patients accepted doctors’ inquiries. They were encouraged to describe their facial perceptions, such as like numbness, formication and any facial discomfort. Data were collected on all treatments rendered and complications that occurred related to treatment. This study provides follow-up outcome data until March 2022.

### Surgical treatments

The objective of the surgery was to remove the bulky tumor and acquire the pathologic results. All patients underwent a detailed systemic examination before the operation, including a complete clinical history, physical examination, complete blood cell count, liver and kidney function tests, chest radiography, and ultrasonography of the neck and abdomen. The routine cardiac examination consisted of a twelve-lead electrocardiogram and echocardiography. Bone marrow aspiration was performed to confirm that there was no bone marrow metastasis. A standard maximal safe resection was performed and meticulous hemostasis was achieved under general anesthesia. All visible tumors were removed during the operation. The tumor specimens were fixed in 10% formaldehyde solution immediately after surgery and sent to the Department of Pathology for pathologic diagnosis.

### Orbital ^125^I seed implantation

After the pathologic diagnosis was established, ^125^I seeds were implanted during the second surgical procedure. The ^125^I radioactive seeds were purchased from Tianjin Saide Medicine Co., Ltd. (Tianjin, China). The ^125^I seed source was a sealed source for radionuclide. The parameters of an ^125^I seed were as follows: activity, 0.5–0.6 mCi; volume, 4.5 mm × Φ 0.8 mm (cylinder); energy, 27–35 keV gamma source; half-life, 59.6 days; tissue penetration, 1.7 cm; half value layer, 0.025 mm lead; source radiation activity, 11.1∼37.9 MBq, and the seed surface was covered with titanium alloy. To prepare the seed tube, individual ^125^I brachytherapy seeds were placed into a peripherally inserted central catheter (PICC), which is a polythene tube with a diameter of approximately 1 mm. The ends of the tubes were sealed so that the ^125^I seeds in the tubes did not move but could be dislodged in the future if necessary. Every tube included 6–8 seeds and the total number of seeds, which determines the brachytherapy dose, was determined by the size of the resected tumor. The number of seeds was calculated using the following formula: seed number = (tumor length + width + height)/3 × 5 ÷ 0.5 mCi [23]. Every seed tube was permanently placed into the nasolacrimal canal or/and under the orbital periosteum around the resection cavity. Tight suture on the orbital periosteum, orbital diaphragm, and skin was achieved after the implantation. All team members wore lead vests and thyroid shields, lead gloves, and protective glasses.

### Statistical analysis

Data were collected and stored with an Excel database (Microsoft Corp., Redmond, WA, USA). Quantitative variables are expressed as the mean ± standard deviation (SD).

## Results

A summary of the demographic characteristics is shown in Table 1. The ages ranged from 49 to 79 years, and the mean age was 60.10 ± 9.16 years. There were eight males and two females. Nine patients had good visual acuity, normal intraocular pressure (IOP), and a normal fundus. One patient had poor visual acuity because of a severe corneal ulcer. One patient had group II cervical lymph nodes and parotid metastasis. The clinical signs and symptoms included proptosis (7 patients), local orbital mass (2 patients), epiphora (1 patient), chemosis (2 patients), eyelid swelling (3 patients), decreased visual acuity (1 patient), ptosis (1 patient), pain (2 patients), and squinting (1 patient). Of 10 patients, 7 were diagnosed clinically based on orbital imaging and a general physical examination with an orbital tumor without metastasis and underwent maximal safe tumor resection at the primary surgery; the pathologic diagnoses were primary orbital lymphomas. ^125^I brachytherapy implantation was carried out for these seven patients at the time of the secondary surgery. None of the seven patients received external radiation or chemotherapy before or after the operation. Three patients had tumor recurrences 6 months after surgery and chemotherapy. Of the remaining 3 patients, one had tumor recurrence at the same location and group II cervical lymph nodes and parotid metastasis 3 months after resection and chemotherapy (patient No. 5). One patient had a tumor recurrence based on a CT scan two months after the first resection and chemotherapy (patient No. 9). One patient was evaluated in the outpatient department because of severe pain and reduced vision; tumor recurrence was demonstrated to occupy the entire left orbital cavity and a corneal ulcer caused by tumor-induced lagophthalmos (patient No. 7). The three patients underwent a second tumor resection combined with ^125^I seed implantation, but no additional chemotherapy before or after implantation.

**Table 1:** Clinical characteristics of the patients

Case	Sex	Age (year)	Visual acuity	IOP (mm Hg)	Fundus	Clinical signs and symptoms
1	Male	62	20/25	12.6	Normal	Proptosis; local orbital mass
2	Male	60	20/25	10.0	Normal	Proptosis; local orbital mass
3	Male	49	20/20	16.1	Normal	Epiphora
4	Male	66	20/20	15.3	Normal	Proptosis; chemosis; pain
5	Male	79	20/20	12.8	Normal	Proptosis; eyelid swelling
6	Male	50	20/20	14.2	Normal	Squint; local orbital mass
7	Female	62	CF	Tn	NA	Pain; chemosis; decreased visual acuity; proptosis; eyelid swelling
8	Male	50	20/20	7.4	Normal	Proptosis
9	Male	58	20/20	18.1	Normal	Proptosis; eyelid swelling
10	Female	65	20/60	9.5	Normal	Proptosis; ptosis

*CF* counting fingers

Individual case data, including tumor size and location, TNM classification, pathologic types, ^125^I brachytherapy seed number, and radiation dose, are summarized in Table 2. All 10 cases were B-cell non-Hodgkin undefined lymphomas. There were six cases of extranodal marginal zone lymphomas of mucosa-associated lymphoid tissue (MALT), one case of small lymphocytic lymphoma, one case of diffuse large B-cell lymphoma, and two cases of mantle cell lymphoma. The TNM staging system showed the following: three cases were T3N0M0; six cases were T4N0M0; and one case was T3N1M0. The tumor was white or red without capsule and adhered closely to the surrounding tissue. The size of the tumor ranged from 1.2 to 6.5 cm. The location of the tumor included the lacrimal gland region (3 cases), deep orbital region adjacent to the optic nerve (2 cases), whole orbital region (1 case), dacryocyst (1 case), and extraocular muscles (3 cases). The number of seeds implanted ranged from 16 to 40.

**Table 2:** Individual case data of tumor information and ^125^I brachytherapy implantation

Case	Tumor location	Tumor size (cm)	Pathology	TNM	Strength (mCi)	Seed number
1	Right infrarectus	2.5 × 1.5	MALT	T4N0M0	0.5	28
2	Right superior rectus	1.5 × 1.5	SLL	T3N0M0	0.5	24
3	Right dacryocyst	1.5 × 2.0	MCL	T3N0M0	0.6	16
4	Left deep orbital region aside optic nerve	2.5 × 1.2	MALT	T3N0M0	0.5	20
5	Left lacrimal gland region and upper forniceal conjunctiva	1.5 × 1.5	MCL	T3N1M0	0.5	18
6	Light infrarectus	2.0 × 3.0	MALT	T4N0M0	0.6	24
7	Left whole orbital region	5.0 × 6.5	MALT	T4N0M0	0.6	40
8	Left lacrimal gland region	3.5 × 3.5	DLBCL	T4N0M0	0.6	32
9	Right deep orbital region aside optic nerve	4.0 × 5.5	MALT	T4N0M0	0.5	40
10	Right lacrimal gland region	4.0 × 3.0	MALT	T4N0M0	0.6	24

*MALT* mucosa-associated lymphoid tissue lymphoma, *SLL* small lymphocytic lymphoma, *MCL* mantle cell lymphoma, *DLBCL* diffuse large B-cell lymphoma

**Fig. 1:**
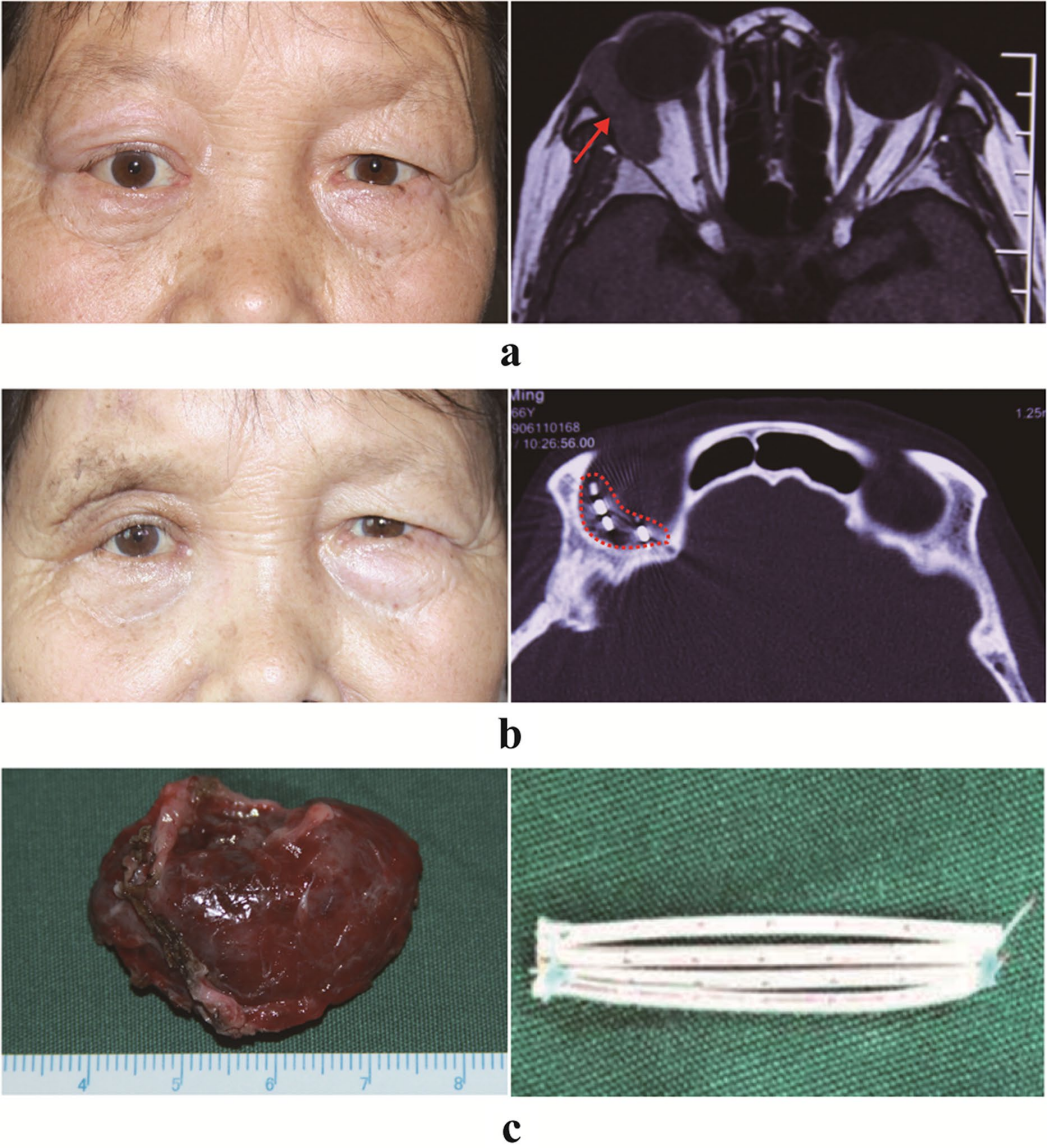
Pre- and postoperative images of patient A. **a** Preoperative images of patient A. The photograph of the patient's appearance shows the right eyeball protruding with eyelid swelling. A CT scan shows a soft tissue mass in the right orbit (red arrow) and squeezing the right eyeball. **b** Seventeen months after brachytherapy. The photograph of the patient's appearance shows no proptosis and no periocular radiation dermatitis. The CT scan shows that the I-125 seeds under the orbital periosteum are stable (red dashed box). **c** The tumor was resected and the size was measured. The seed tube is in preparation

**Fig. 2:**
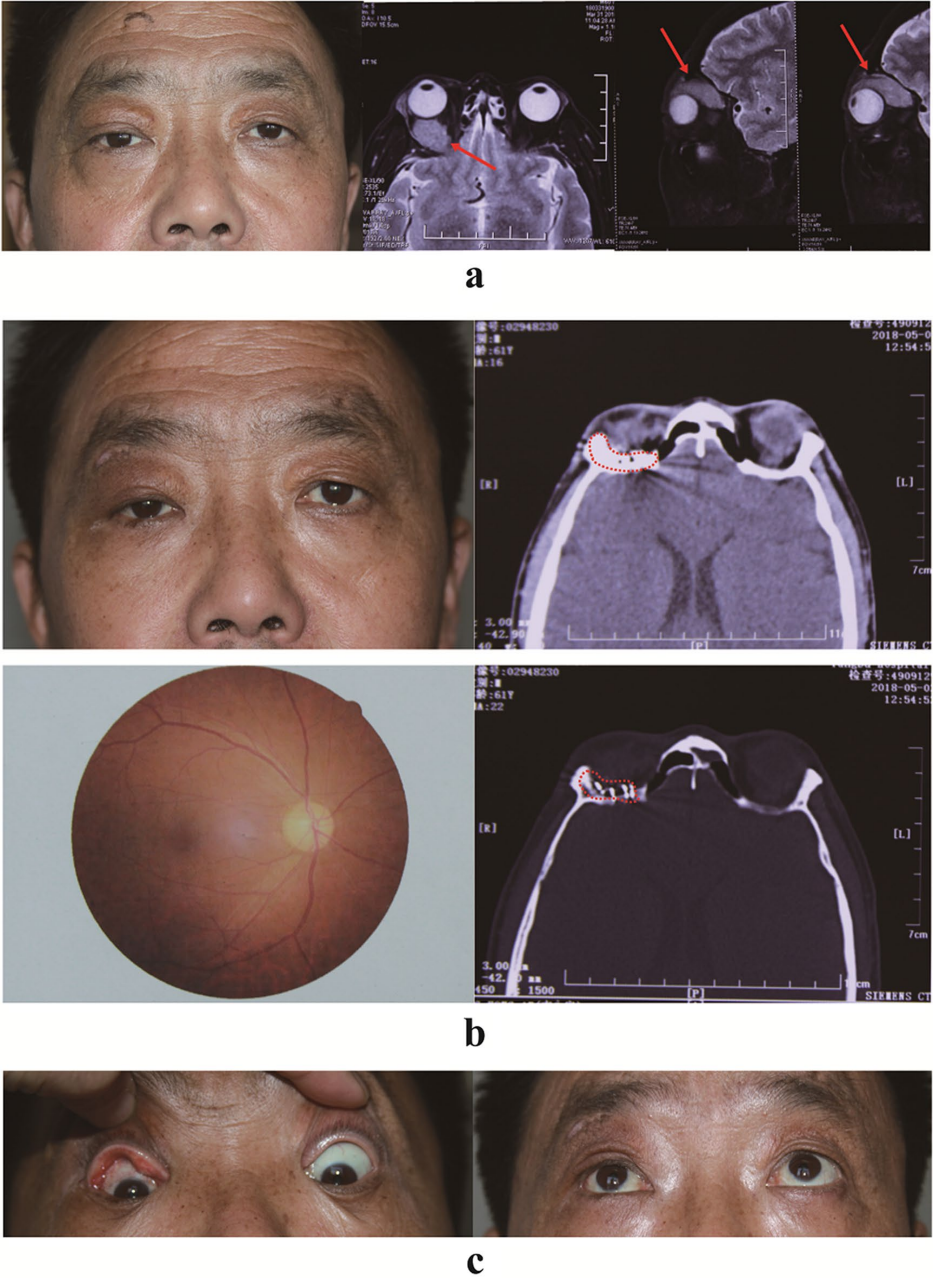
Pre- and postoperative images of patient B. **a** Preoperative images of patient B. The photograph of the patient's appearance shows the right eyeball protruding and fixed downward. A CT scan shows a soft tissue mass in the right superior rectus (red arrows). **b** Twenty-nine months after brachytherapy. The photograph of the patient's appearance shows no proptosis and no periocular radiation dermatitis. The CT scan shows that the I-125 seeds under the orbital periosteum are stable (red dashed boxes). The fundus photograph of the right eye was normal. **c** Eye movements (upward and downward) return to normal

**Fig. 3:**
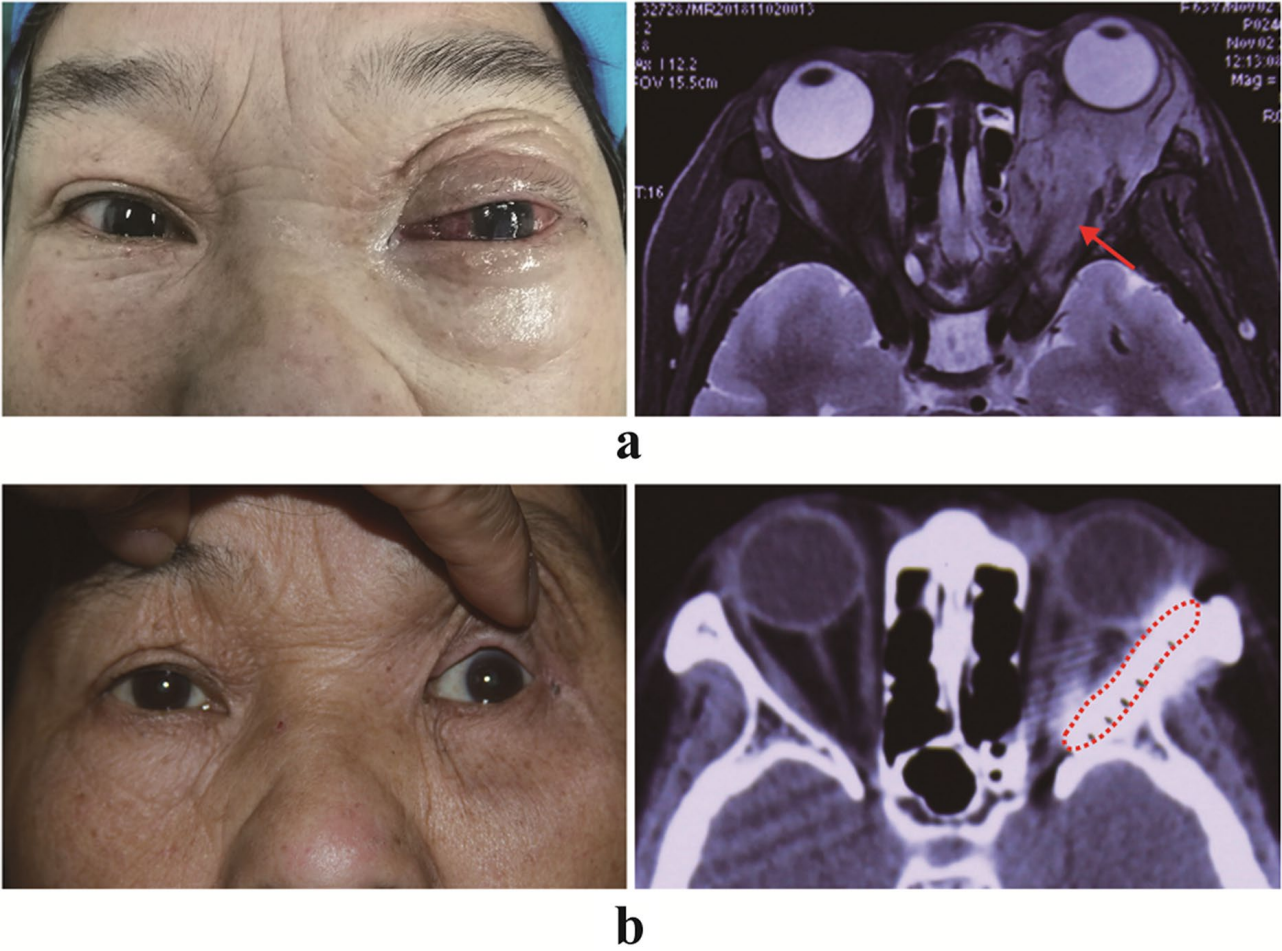
Pre- and postoperative images of patient C. **a** Preoperative images of patient C The photograph of the patient's appearance shows the left eyeball protruding and a severe corneal ulcer. A CT scan shows a large soft tissue mass in the left whole orbital region (red arrow). **b** Twenty months after brachytherapy. The photograph of the patient's appearance shows no proptosis and no periocular radiation dermatitis. The left cornea became clean, and the patient’s vision significantly improved. The CT scan shows that the I-125 seeds under the orbital periosteum are stable (red dashed box)

The follow-up outcome data are shown in Table 3 and the follow-up period ranged from 40 to 65 months. All patients in this study are alive and well with good tumor control (Figs. 1, 2). Four patients underwent partial tumor resections because the tumor was too large to completely remove and was closely adherent to the surrounding tissues. Fortunately, the tumor regressed 3 months after implantation in all 4 patients. The patient with decreased vision secondary to the tumor-induced severe corneal ulcer had improved vision and the corneal ulcer resolved 1 month after implantation (Fig. 3). The primary clinical signs and symptoms, including proptosis, epiphora, eyelid swelling, pain, and squinting, completely resolved. No patient had tumor recurrence or metastasis after implantation during the follow-up period. No patient had radiodermatitis involving the skin around the eye. No patient had radiation-related ophthalmopathy, including glaucoma, cataract, retinopathy, or papillopathy. Only three patients had mild dry eye symptoms and two patients had abnormal facial sensations.

**Table 3:** Follow-up data after ^125^I brachytherapy implantation

Case	Follow-up (month)	Systemic status	Tumor recurrence	Tumor metastasis	Eye status	Complications
1	65	Alive and well	No	No	Intact	Abnormal facial sensation
2	55	Alive and well	No	No	Intact	No
3	63	Alive and well	No	No	Intact	No
4	61	Alive and well	No	No	Intact	No
5	57	Alive and well	No	No	Intact	Dry eye syndrome
6	59	Alive and well	No	No	Intact	No
7	56	Alive and well	No	No	Vision improvement	Dry eye syndrome
8	55	Alive and well	No	No	Intact	Dry eye syndrome
9	40	Alive and well	No	No	Intact	Abnormal facial sensation
10	40	Alive and well	No	No	Intact	No

## Discussion

Orbital lymphoma is one of the most common adult orbital malignancy, accounting for 10% of all orbital tumors. The vast majority of orbital lymphomas are of B-cell origin (97%), of which MALT lymphoma is the most common subtype (59%), followed by diffuse large B-cell lymphoma (DLBL [23%]), follicular lymphoma (FL [9%]), and mantle cell lymphoma (MCL [5%]) [24]. MALT and FL are low-grade lymphomas that have a good prognosis, whereas high-grade lymphomas, such as DLBL and MCL, are associated with a poor prognosis. In our study, patients with different subtypes of the lymphomas had a 100% survival rate without local recurrence or metastasis.

External radiotherapy (ER) is frequently used in the treatment of orbital lymphomas; however, it is associated with treatment toxicity. It has been reported that > 90% of patients who undergo ER develop grade-one or -two dry eye that requires long-term treatment. Radiation cataracts are also common during ER treatment. It has been reported that one-half of patients have radiation cataracts and need cataract extraction [25]. In addition, keratitis and macular degeneration are rare complications of ER [10]. In the current study, an ophthalmologic examination, including vision, IOP, lens, and fundus, was performed on all patients before and after treatment and at every follow-up visit. No patients had ophthalmologic complications, likely for several reasons. Unlike X-rays used for ER, the ^125^I brachytherapy mainly takes the form of X-rays, which have lower penetrating power. ^125^I brachytherapy radiation primarily kills tumor cells that proliferate faster, but causes no apparent damage to eye tissues or optic nerve cells, which are in the relatively stationary phase [23, 26]. The effective emission distance of the ^125^I seeds was only 13 mm and we implanted the seeds under the periosteum. Consequently, some of the radiation from the seeds is absorbed by orbital bone, and the other half acts on the cavity after removal of the tumor tissue, possibly killing residual tumor cells. Because of the short radiation distance of the seed, the seed does not cause damage to normal tissues, such as the eye, subcutaneous tissue, and skin. We chose the nasolacrimal canal or/and subperiosteum to implant seed tubes because they are the perfect places to both ensure radiation effectiveness to residual tumor cells and also protects the eye tissues from intense radiation damage. In another case (data not shown), a patient had whole sclera invasion by the tumor. After maximal removal of the tumor, we tried to fix seed tubes on the sclera to eradicate residual tumor cells. Unfortunately, 2 months later, this patient had severe radiation aseptic endophthalmitis, and enucleation was performed.

To calculate the number of ^125^I seeds to implant, we first estimated the tumor size based on the preoperative imaging results and then calculated the actual number of seeds using the formula mentioned in the method according to practical measurements of tumor size after resection. Thus, the number of ^125^I seeds that were actually implanted into the tumor was, in theory, the number of seeds that were implanted directly into the tumor body without having to remove the tumor. We chose this number of seeds to implant despite the resection of tumor during the primary surgery in order to ensure the effectiveness of ^125^I brachytherapy, considering that the seeds were implanted into the nasolacrimal canal or/and under the periosteum and greater than one-half of the radiation dose would be absorbed by bone tissue.

During the operation, we placed 6–8 seeds into the PICC one-by-one and implanted the seed tube under the periosteum. We chose the PICC for the following reasons. First, the PICC is a safe, tissue-implanted tube that does not damage tissues and can be implanted in the human body for a long time. Second, we found that the PICC material had no effect on ^125^I seed radioactivity. In primary experiments, we measured the radioactivity of individual seeds placed outside and inside the PICC and found no apparent change in radioactivity. Third, scattered seeds are easily dissociated in the tissue over time or attracted by MRI and result in local tissue damage. In a former case, the location of implanted seed removal was demonstrated, and the seeds were very close to the optic nerve. Fourth, the seed tube is easy to find and remove or replace if the radiation is depleted or exposed to tissues.

In some studies, a three-dimensional radiation therapy planning system (TPS) was applied before brachytherapy. In the current study, we did not use TPS preoperatively. TPS is mainly used for the preoperative evaluation of the brachytherapy dose and seed implantation position for unresectable solid tumors. In our study, tumor was removed before seed implantation and the purpose of brachytherapy was postoperative prevention of tumor recurrence and metastasis. In addition, the number of seeds implanted was calculated using the formula based on the tumor size, which is measured directly after tumor resection during surgery.

When patients only have local tumor and excellent eye conditions, external radiation may be undesirable, particularly since a variety of complications may affect their quality of life. Hence, surgical resection with orbital ^125^I brachytherapy appears to be a legitimate therapeutic alternative. In conclusion, orbital ^125^I brachytherapy seed implantation is a novel and effective method for the treatment of local lymphomas. As a safe and effective treatment method with the advantages of a simpler operation, smaller intraoperative injury and fewer complications, orbital ^125^I brachytherapy seed implantation is worth applying in the treatment of orbital malignancies. The limitations of the study are the smaller sample size and shorter follow-up time. In the later stage, it is still necessary to expand the large sample size and observe whether the mass has recurrence or metastasis after long-term follow-up. Further comparative studies with the effect of systemic chemotherapy will be conducted.
